# Synergistic Antibacterial and Anti-inflammatory Activities of *Ocimum tenuiflorum* Ethanolic Extract against Major Bacterial Mastitis Pathogens

**DOI:** 10.3390/antibiotics11040510

**Published:** 2022-04-12

**Authors:** Janejira Srichok, Natthika Yingbun, Teerada Kowawisetsut, Sudsaijai Kornmatitsuk, Uthaiwan Suttisansanee, Piya Temviriyanukul, Boonrat Chantong

**Affiliations:** 1 Faculty of Veterinary Science, Mahidol University, Salaya, Phutthamonthon, Nakhon Pathom 73170, Thailand; janejira.srichok@gmail.com (J.S.); natthika.yingbun@gmail.com (N.Y.); teerada.kow@gmail.com (T.K.); 2 Department of Clinical Science and Public Health, Faculty of Veterinary Science, Mahidol University, Salaya, Phuttamonthon, Nakhon Pathom 73170, Thailand; sudsaijai.kor@mahidol.ac.th; 3 Food and Nutrition Academic and Research Cluster, Institute of Nutrition, Mahidol University, Salaya, Phuttamonthon, Nakhon Pathom 73170, Thailand; uthaiwan.sut@mahidol.ac.th (U.S.); piya.tem@mahidol.ac.th (P.T.); 4 Department of Pre-Clinical and Applied Animal Science, Faculty of Veterinary Science, Mahidol University, Salaya, Phuttamonthon, Nakhon Pathom 73170, Thailand

**Keywords:** antibacterial activity, anti-inflammation, mastitis, *Ocimum tenuiflorum* L., synergistic

## Abstract

Mastitis is the most prevalent global illness affecting dairy cows. This bacterial infection damages and inflames the udder tissues. Several plant extracts have demonstrated synergistic antibacterial activities with standard drugs in mastitis treatment. Scant information exists on *Ocimum tenuiflorum* L. This study evaluated the antibacterial activity of *O. tenuiflorum* extract and its interaction with antibacterial drugs against common mastitis pathogens including *Staphylococcus aureus*, coagulase-negative Staphylococci (CNS), *Streptococcus agalactiae*, and *Escherichia coli*. Anti-inflammatory activities in LPS-stimulated RAW264.7 macrophage cells were also studied. The *O. tenuiflorum* extract exhibited antibacterial activities against *S. aureus*, CNS, and *S. agalactiae* with minimum inhibitory concentration (MIC) ranging from 3.9 to 31.2 µg/mL and minimum bactericidal concentration (MBC) ranging from 15.6 to 500 µg/mL. Combinations of *O. tenuiflorum* with penicillin or amoxicillin-clavulanic acid showed synergistic effects against all tested strains but an additive effect with cefazolin and gentamicin. Pretreatment of the extract significantly decreased the expression of inflammatory molecules (IL-6, TNF-α, IL-1β, iNOS, COX-2, and PGE2) generated by LPS in macrophages. Results suggested *O. tenuiflorum* effectiveness against various Gram-positive mastitis bacteria, with the potential to reduce antibacterial doses and combat inflammation.

## 1. Introduction

Bovine mastitis is an inflammatory condition of the udder, most often caused by bacterial intramammary infection and resulting in significant economic losses of milk from dairy cattle [1]. Mastitis is typically associated with bacterial infection, brought on by inadequate cattle management, housing, and milking [2]. Primary bacteria responsible for mastitis in dairy cows are *Staphylococcus aureus* (*S. aureus*), *Streptococcus agalactiae* (*S. agalactiae*), *Escherichia coli* (*E. coli*), *Enterococcus* spp., and coagulase-negative Staphylococci (CNS) [3,4]. In Thailand, the most frequently isolated bacteria involved in clinical and subclinical mastitis are *E. coli*, *Streptococcus* spp., and *Staphylococcus* spp. [5,6,7,8,9]. Antibacterial treatment is a well-established concept in mastitis management but often leads to the emergence of antibiotic-resistant bacteria [2,10]. Infectious multidrug-resistant bacteria have become a serious public health problem for both human and animal populations [11]. Antibiotics used to treat bovine mastitis have been identified as a frequent source of drug residues in milk [10]. Therefore, alternative approaches are required for mastitis management in dairy cows. Antibacterials derived from plants have no effect on resistance development in Gram-positive and Gram-negative bacteria following prolonged exposure [3,10,12]. Plant extracts have recently shown promise as synergistic promoters. Antibacterial drugs in combination with plant extracts extended the antibacterial range while reducing antibacterial doses [13,14].

During mastitis, the mammary immune microenvironment produces cytokines and other pro-inflammatory mediators in nearby cells such as macrophages [15,16,17]. Cytokine levels as tumor necrosis factor-α (TNF-α), interleukin-1β (IL-1β), interleukin-6 (IL-6), and interleukin-8 (IL-8) as well as other molecules including nitric oxide (NO) are elevated during infections [18,19,20,21]. Cyclooxygenase-2 (COX-2) is an enzyme involved in the conversion of arachidonic acid into prostaglandin E2 (PGE2), which produces fever and inflammation in the body is also up-regulated during mastitis [22].

Lipopolysaccharide (LPS) forms part of the outer membrane of Gram-negative bacteria and is recognized as a virulence factor for bovine mastitis [23]. LPS stimulates the production of cytokines in the host, thereby initiating an inflammatory response, and is frequently used in animal models to promote mastitis. Supporting animal health by promoting natural immunological defenses with immunomodulating agents offers an interesting additional supplement to antibacterial treatment [24] using natural compounds to regulate cytokine production. Morin from *Morus* spp. had a protective effect against LPS-induced mastitis, probably related to its anti-inflammatory activities, by inhibiting the NLRP3 inflammasome and the NF-κB signaling pathway [20]. The essential oil derived from plants suppressed pro-inflammatory cytokine production in response to bacterial inflammation [21]. In bovine subclinical mastitis, a topical mixed herbal gel decreased bacterial load and modulated somatic cell cytokine expression [25]. Thus, anti-inflammatory mechanisms can be considered in mastitis treatment to support antibacterial properties.

Holy basil (*Ocimum tenuiflorum* L.) is a medicinal herb in the Lamiaceae family that grows in tropical regions. This plant has essential volatile oil comprising phenols, terpenes, and aldehydes that are mainly concentrated in the leaf [26,27]. Evidence suggested *O. tenuiflorum* is beneficial in treating mastitis. An intramammary administration of *O. tenuiflorum* aqueous extract decreased total bacterial count, elevated neutrophil and lymphocyte counts, and lysosomal enzyme content of milk polymorphonuclear cells [28], while a hydro-alcoholic extract of *O. tenuiflorum* leaves showed antibacterial properties against common bovine mastitis pathogens such as *S. aureus*, CNS, *Streptococcus* spp., *E. coli*, *Klebsiella* spp. and *Corynebacteriam* spp. [29]. *S. aureus*, including methicillin-resistant *S. aureus* (MRSA) and *E. coli*, were inhibited by essential oil isolated from *O. tenuiflorum* [26].

In addition, *O. tenuiflorum* has been studied for its anti-inflammatory effects. The aqueous extract was able to reduce the cytotoxicity of lung epithelial cells infected with Klebsiella pneumoniae by decreasing the quantity of pro-inflammatory cytokines [30]. The ability of Basil to reduce the expression of the nuclear factor-kappaB (NF-κB) was considered to be the mechanism of its anti-inflammatory properties [30,31]. However, the impact of *O. tenuiflorum* against clinically isolated pathogen-related mastitis in combination with antibacterial drugs and its anti-inflammatory properties remains elusive. Thus, this study assessed the antibacterial activity and potentiation effect of *O. tenuiflorum* against common mastitis pathogens including *S. aureus*, CNS, *S. agalactiae* and *E. coli* strains from the American Type Culture Collection (ATCC) and field-collected bacteria. The anti-inflammatory activities of *O. tenuiflorum* in LPS-stimulated RAW264.7 macrophages were also examined. Results showed that *O. tenuiflorum* exhibited antibacterial activity against Gram-positive pathogenic organisms, enhanced the antibacterial effect of bovine mastitis drugs, and possessed anti-inflammatory properties. This study forms the basis for subsequent in vitro and in vivo investigations of *O. tenuiflorum* to reveal new phytochemical components for the treatment of mastitis.

## 2. Results

### 2.1. Qualitative Phytochemical Analysis of Ethanolic Extract of O. tenuiflorum

**Figure 1:**
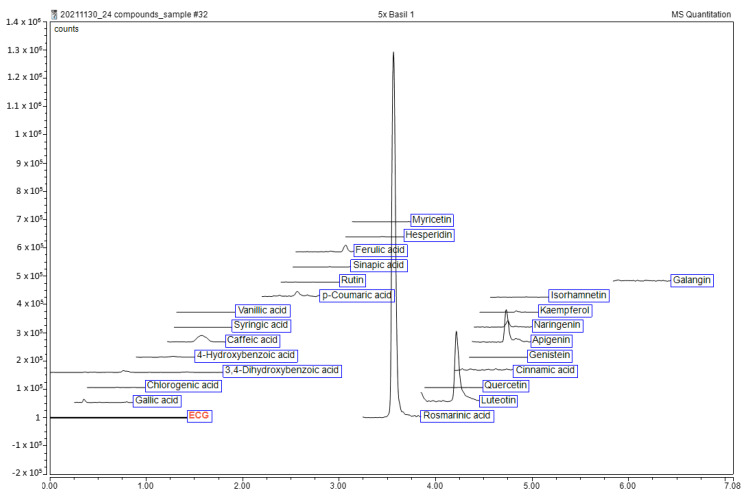
Liquid chromatography-electrospray ionization tandem mass spectrometry (LC-ESI-MS/MS) chromatogram of ethanol extract of *O. tenuiflorum*.

Leaves of *O. tenuiflorum* were extracted with 70% ethanol, yielding 7.65% *w*/*w* (based on dried plant material). The ethanolic extract of *O. tenuiflorum* was then assessed for its phytochemical constituents by liquid chromatography-electrospray ionization tandem mass spectrometry (LC-ESI-MS/MS). Chromatograms and retention times against 23 authentic standards are shown in Figure 1. Three compounds were reported including rosmarinic acid (147.54 ± 1.79 mg/100 g extract), luteolin (22.7 ± 2.46 mg/100 g extract) and apigenin (31.55 ± 2.82 mg/100 g extract).

### 2.2. MIC and MBC of the Antibacterial Drugs and O. tenuiflorum Extract

In vitro antibacterial activity, along with a minimum inhibitory concentration (MIC) and minimum bactericidal concentration (MBC) of antibacterial drugs commonly used in the treatment of mastitis and *O. tenuiflorum* ethanol crude extracts were tested against bacterial mastitis pathogens including *S. aureus* (ATCC25923, clinical isolate), CNS (clinical isolate), *S. agalactiae* (ATCC17129, clinical isolate) and *E. coli* (ATCC25922, clinical isolate), as shown in Table 1. DMSO at 0.5% used as a solvent control showed no inhibitory effect. MIC values of penicillin, amoxicillin-clavulanic acid, cefazolin, and gentamicin against *S. aureus* and *S. agalactiae* obtained in the field were greater than the values of the reference strains, while *E. coli* susceptibility to gentamicin was similar across the standard and clinical strains at MIC of 2 µg/mL.

In vitro susceptibility tests showed that *O. tenuiflorum* extract had antibacterial effects against Gram-positive bacteria including *S. aureus*, CNS, and *S. agalactiae* but not against *E. coli* Gram-negative bacteria, with MICs ranging from 3.9 to 31.2 µg/mL. Similar to the antibiotic susceptibility results, the standard strains were more susceptible to the extract than the clinical stains. The extract showed MBC values of 62.5, 125, 15.6, 125, and 500 µg/mL against *S. aureus* (ATCC25923), *S. aureus* (clinical isolate), CNS (clinical isolate), *S. agalactiae* (ATCC17129) and *S. agalactiae* (clinical isolate), respectively. The MBC/MIC ratio indicates antibacterial activity. A value less than or equal to four is considered bactericidal, while at a value of more than four the action is considered bacteriostatic [32]. The most promising activity was shown against *S. aureus* (ATCC25923; clinical isolate) and CNS, with MBC/MIC values of four indicating bactericidal activity. Findings demonstrated that *O. tenuiflorum* extract showed promising activity against some Gram-positive bacteria from prevalent veterinary mastitis pathogens, with standard strains more susceptible than clinical isolates.

### 2.3. Evaluation of the Synergistic Effect of Antibacterial Drugs and O. tenuiflorum Extract

**Figure 2:**
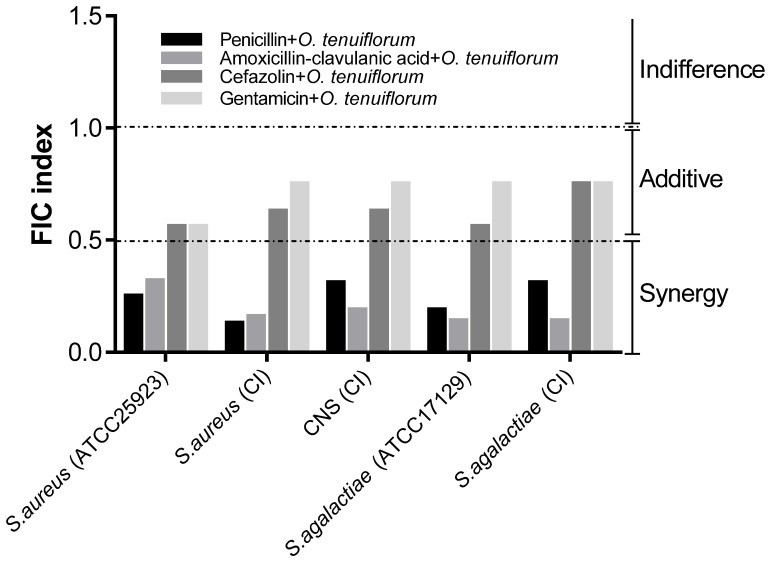
Fractional inhibitory concentration (FIC) index of bacterial strains for antibacterial drugs and *O. tenuiflorum* extract combinations. CI = clinical isolate.

A checkerboard assay was used to determine the interaction between antibacterial drugs and the *O. tenuiflorum* extract. Multiple dilutions of antibacterial drugs were combined with a variety of extract concentrations. A fractional inhibitory concentration (FIC) index was calculated for each tested combination against *S. aureus* (ATCC25923; clinical isolate), CNS (clinical isolate), and *S. agalactiae* (ATCC17129; clinical isolate), as shown in Figure 2. The combination of penicillin, amoxicillin-clavulanic acid, and *O. tenuiflorum* extract demonstrated synergistic benefits against all tested strains, with extract FIC index values less than or equal to 0.5, while the action of cefazolin and amikacin in combination with the extract demonstrated an additive effect. Antibacterial drugs and the extract interacted similarly with standard and clinical strains. Results suggested that *O. tenuiflorum* extract could be utilized to significantly reduce concentrations of antibacterial drugs.

### 2.4. Cytotoxicity Assay with RAW 264.7 Murine Macrophage Cells

**Figure 3:**
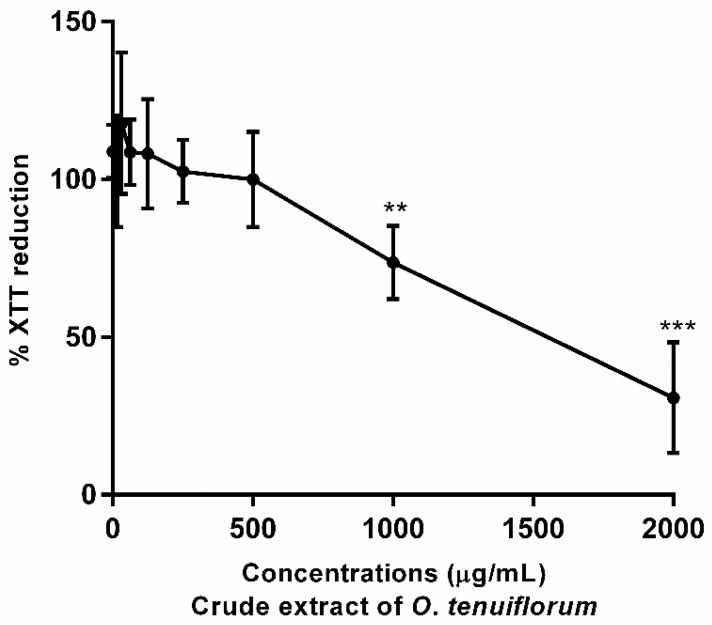
Effects of *O. tenuiflorum* extract RAW264.7 cell viability. Cells were treated with different concentrations (15.625–2000 µg/mL) of *O. tenuiflorum* extract for 24 h, with cell viability determined by XTT reduction assay. Values are presented as the mean ± standard deviation of at least three replicates. **, *p* < 0.01; ***, *p* < 0.0005 vs. the control.

The XTT reduction assay was employed to determine the effect of *O. tenuiflorum* extract on cell viability in macrophage cells. The *O. tenuiflorum* extract exhibited a low level of cytotoxicity that subsequently increased in a concentration-dependent manner to an IC_50_ of 1000 µg/mL in RAW 264.7 cells (Figure 3). No cytotoxicity was observed in RAW 264.7 cells for MICs and MBCs against *S. aureus*, CNS and *S. agalactiae*. The *O. tenuiflorum* extract was diluted and tested in the presence of 0.5% (*v*/*v*) DMSO (final concentration), with no toxic effects on RAW 264.7 cells (data not shown). The extract at 500 µg/mL was not cytotoxic to RAW 264.7 cells, and this concentration was selected for assessment of subsequent anti-inflammatory properties.

### 2.5. O. tenuiflorum Extract Suppressed LPS-Induced Expression of Pro-Inflammatory Cytokines in RAW 264.7

**Figure 4:**
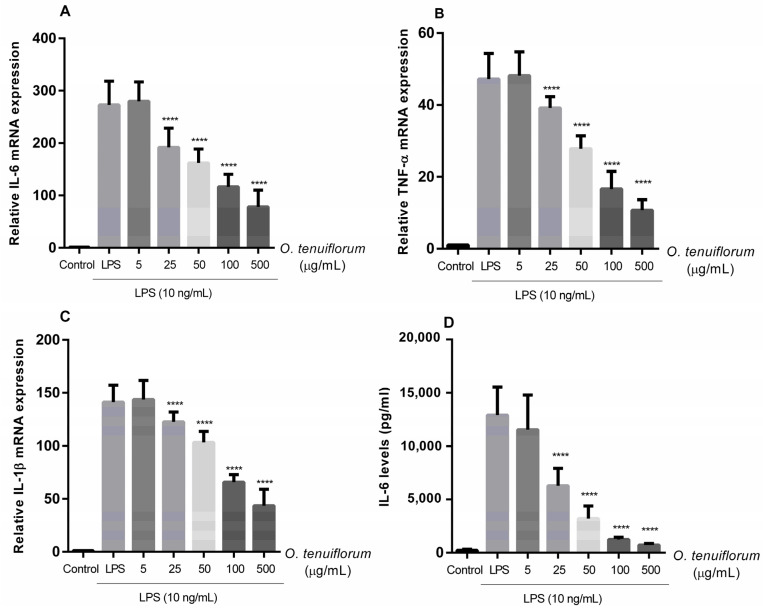
Effects of *O. tenuiflorum* extract on LPS-induced pro-inflammatory cytokine expression in RAW 264.7. (**A**–**C**) Cells were not treated or pretreated with *O. tenuiflorum* extract (5, 25, 50, 100, or 500 µg/mL) for 12 h before stimulation with LPS (10 ng/mL) for 24 h. The control cells were not treated with LPS or *O. tenuiflorum*. Total RNA was prepared from cells and the mRNA levels of IL-6, TNF-α, and IL-1β were determined by qRT-PCR, as described in Materials and Methods. (**D**) IL-6 production in the culture media was quantified using an ELISA kit. Values are presented as the mean ± standard deviation of at least three replicates. ****, *p* < 0.0001 vs. LPS-stimulated cells. LPS, lipopolysaccharide; IL-6, interleukin-6; TNF-α, tumor necrosis factor-α; IL-1β, interleukin-1β.

In mastitis, activated macrophages play an essential role in triggering innate immunity, initiating the pro-inflammatory response through cytokine production, and linking inflammatory tissue [33]. Concentration-dependent suppression in mRNA expression of IL-6, TNF-α, and IL-1β was found with *O. tenuiflorum* pretreatment at concentrations ranging from 25 to 500 µg/mL in LPS-activated RAW264.7 cells (Figure 4A–C). Similar to mRNA expression levels, the amount of IL-6 induced by LPS significantly reduced in cells pretreated with *O. tenuiflorum* (Figure 4D). These findings indicated that pretreatment with *O. tenuiflorum* significantly reduced an exaggerated immune response in RAW 264.7 macrophages by inhibiting the expression of pro-inflammatory cytokines induced by LPS.

### 2.6. O. tenuiflorum Extract Suppressed LPS-Induced iNOS and COX-2 Expression and NO and PGE2 Production in RAW 264.7 Macrophages

**Figure 5:**
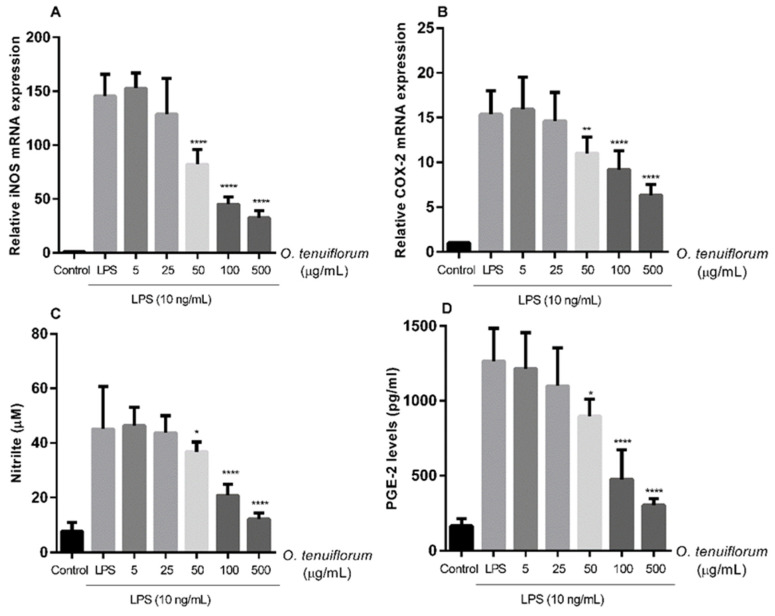
Effects of *O. tenuiflorum* extract on LPS-induced COX-2 and iNOS mRNA expression and NO and PGE2 production in RAW 264.7. (**A**,**B**) Cells were not treated or pretreated with *O. tenuiflorum* extract (5, 25, 50, 100, or 500 µg/mL) for 12 h before stimulation with LPS (10 ng/mL) for 24 h. The control cells were not treated with LPS or *O. tenuiflorum*. Total RNA was prepared from cells and the mRNA levels of iNOS and COX-2 were determined by qRT-PCR, as described in Materials and Methods. (**C**) NO production in the culture media was quantified using the Griess reaction assay. (**D**) PGE2 production in the culture media was quantified using an enzyme-linked immunosorbent assay kit. Values are presented as the mean ± standard deviation of at least three replicates. *, *p* < 0.05; **, *p* < 0.01; ****, *p* < 0.0001 vs. LPS-stimulated cells. LPS, lipopolysaccharide; iNOS, inducible nitric oxide synthase; COX-2, cyclooxygenase-2; NO, nitric oxide; PGE2, prostaglandin E2.

In addition to cytokines, various inflammatory chemicals are released during mastitis infection and contribute to local and systemic inflammation [19,20,34,35]. qPCR was used to determine changes in iNOS and COX-2 transcription and assess the anti-inflammatory properties of *O. tenuiflorum* extract, while the Griess assay and ELISA were used to quantify NO and PGE2 concentrations released in cell culture supernatants after *O. tenuiflorum* pretreatment. To evaluate the inhibitory effects of *O. tenuiflorum* on LPS-induced iNOS and COX-2 upregulation, cells were treated with *O. tenuiflorum* at concentrations of 5, 25, 50, 100, or 500 g/mL for 12 h followed by 24 h of stimulation with LPS (10 ng/mL). *O. tenuiflorum* decreased LPS-stimulated expression of iNOS and COX-2 in a concentration-dependent manner (Figure 5A,B). The effect was first observed at an extract concentration of 50 µg/mL, whereas suppression of cytokine expression was initially seen at an extract concentration of 25 µg/mL (Figure 4A–D). Pretreatment with *O. tenuiflorum* resulted in a significant reduction in NO production, which was associated with a decrease in iNOS mRNA expression (Figure 5C). PGE2 levels were assessed after macrophages were exposed to *O. tenuiflorum* at the indicated concentrations. Likewise, *O. tenuiflorum* pretreatment reduced LPS-induced PGE2 production in a concentration-dependent manner (Figure 5D). These findings showed that *O. tenuiflorum* had an inhibitory effect on LPS-induced stimulation of RAW 264.7 macrophages by suppressing inflammation-mediated consequences.

## 3. Discussion

Mastitis, the most commonly occurring bacterial infection in dairy cows, is a type of breast tissue infection, characterized by alterations in the mammary tissues and inflammation [1]. The standard approach to treating the disease in dairy farms is using antibacterial agents such as penicillin, amoxicillin-clavulanic acid, cefazolin, or gentamicin. However, bacterial resistance continues to develop and expand and antibacterial alternatives for mastitis prevention and treatment are of great interest worldwide [10,11]. Natural substances found in medicinal plants have been intensively explored as potential antibacterial agents to reduce the complications associated with antibacterial-resistant bacteria and drug residues in milk [12]. For instance, the essential oils of *Thymus serpyllum* and *Thymus vulgaris* have shown promising antibacterial efficiency against mastitis-associated bacteria and certain antioxidative activities, suggesting that these might be used as an alternative treatment in mastitis [36]. The combination with a phenolic extract from *Eucalyptus globulus* and penicillin G exhibited synergy against *S. aureus* [37]. The combination of extracts from *Syzygium aromaticum*, *Cinnamomum verum*, *Emblica officinalis*, *Terminalia belerica*, *Terminalia chebula*, and *Cymbopogon citratus* exhibited remarkable antibacterial and antibiofilm activities against clinical isolates and a reduction in the virulence factors [38]. *O. tenuiflorum*, a common herbal plant in Southeast Asia, has shown promise in treating mastitis [26,28,29]. However, the antibacterial efficacy of *O. tenuiflorum* against clinically isolated pathogen-related mastitis and combination effects with antibacterial drugs remains unknown. Furthermore, inflammation observed by the induction of inflammatory mediators also occurs during mastitis. Thus, to support antibacterial properties, anti-inflammatory compounds can also be considered in mastitis treatment. This study evaluated the antibacterial activity and synergistic effects of *O. tenuiflorum* extract against prevalent mastitis pathogens including *S. aureus*, CNS, *S. agalactiae* and *E. coli.* The anti-inflammatory activities of *O. tenuiflorum* extract were evaluated using LPS-stimulated RAW264.7 murine macrophage cells as a model.

Clinical strains of *S. aureus* and *S. agalactiae* were less susceptible to antibacterial drugs tested as compared to standard stains. The prevalence and susceptibility patterns of mastitis-pathogens reveal considerable regional variation and also significant variances in diverse communities and circumstances [39,40,41]. Continuous screening of antibacterial susceptibility of clinical isolates is suggested and considered suitable for evidence-based mastitis treatment in each location. In Thailand, 46% of *S. aureus* isolated from milk with subclinical mastitis was identified as methicillin-resistant *S. aureus* [8]. The CLSI VET08 guideline defines clinical breakpoints for susceptibility testing for three antibacterial drugs used to treat bovine mastitis, including ceftiofur, penicillin/novobiocin, and pirlimycin. While in Thailand, frequently used antibacterial agents for the management of mastitis are penicillin, amoxicillin-clavulanic acid, and cefazolin. The CLSI recommends a 2:1 amoxicillin-clavulanic acid ratio; however, this research used a 4:1 ratio since this ratio in preparations is routinely used in Thailand (Synulox^®^) [42,43,44].

The antibacterial properties of an ethanolic extract of *O. tenuiflorum* against bacterial strains associated with bovine mastitis are reported here. The antibacterial activity of *O. tenuiflorum* extract against Gram-positive organisms was considerable. The extract showed bactericidal activity against *S. aureus* and CNS. Antibacterial activity of *O. tenuiflorum* against *S. aureus*, CNS, and *E. coli* isolates was reported in India, with MICs higher than ours. The susceptibility of bacteria and bioactive ingredients in the extract vary at each site [29]. A possible target of *O. tenuiflorum* action against Gram-positive bacteria is the cell membrane, involving disruption of phospholipid bilayers based on the extract’s lipophilic characteristics [45,46]. The combination of penicillin and amoxicillin-clavulanic acid plus *O. tenuiflorum* extract had synergistic effects on all studied strains, whereas the combination of cefazolin and gentamicin with plant extract showed a mostly additive effect. Optimal synergistic capacity against bacterial strains was recorded with amoxicillin-clavulanic acid. Proposed mechanisms underlying the synergistic impact of plant extracts include suppression of bacterial protection enzymes, successive inhibition of biochemical pathways, and enhancement of antibacterial diffusion [47].

This study explored the anti-inflammatory properties of *O. tenuiflorum* extract in combination with its antibacterial activity to reduce mastitis-related inflammatory symptoms on LPS-activated RAW 264.7 cells. *O. tenuiflorum* dramatically reduced inflammatory responses in LPS-activated RAW 264.7 macrophages by decreasing IL-6, TNF-α, IL-1β, iNOS, NO, COX-2, and PGE2 expression levels. The pro-inflammatory cytokines NO and PGE2 were significant in the inflammatory process, while bacteria invaded the mammary glands [18,19,20,21,22]. These findings suggested that *O. tenuiflorum* extract reduced tissue damage and protected tissue from bacterial-induced mastitis.

The three major phytochemicals identified in the extract were rosmarinic acid (0.15%), apigenin (0.032%), and luteolin (0.023%), with suggested antibacterial and anti-inflammatory activities. Rosmarinic acid inhibited a key virulence factor on the cell surface, demonstrating antibacterial action and synergistic effects, with antibacterial drugs against *S. aureus* and MRSA [48]. Rosmarinic acid has anti-inflammatory benefits in a variety of disorders, including mastitis [49,50]. Apigenin, a flavonoid phytochemical, showed antibacterial activity against various strains of bacteria including *Enterobacter* spp., *E. coli*, and *Pseudomonas aeruginosa* but had low activity against *S. aureus* [51]. Apigenin, when combined with ampicillin, increased antibacterial action against *Streptococcus suis*, while simultaneously inhibiting cytokine production from infected cells [52]. Luteolin, a hydroxyflavone, showed potent anti-inflammatory properties and decreased the expression of pro-inflammatory cytokines in mammary tissues and epithelial cells infected with *S aureus* [53,54]. The antibacterial properties of luteolin, although not significant, reduced the expression of the antibiotic resistance gene (multidrug and toxic compound extrusion gene) in *Trueperella pyogenes* [55]. Based on these findings, rosmarinic acid, apigenin and luteolin bioactive components in *O. tenuiflorum* ethanolic extract showed antibacterial and anti-inflammatory properties.

## 4. Materials and Methods

### 4.1. Bacterial Strains, Cell Cultures, Chemicals, and Reagents

The standard bacterial strains used in this study were *S. agalactiae* (ATCC17129), *S. aureus* (ATCC25923), and *E. coli* (ATCC25922). They were obtained from Assoc. Prof. Dr. Norasuthi Bangphoomi, Faculty of Veterinary Science, Mahidol University, Thailand. Clinical isolates of *S. aureus*, *S. agalactiae*, coagulase-negative staphylococci (CNS), and *E. coli* isolated from clinical bovine mastitis cases were obtained from milk samples collected from Dairy Farming Promotion Organization of Thailand (D.P.O.). All bacteriological media used in the study were purchased from Oxoid Ltd. (Basingstoke, Hampshire, UK).

Lipopolysaccharide (LPS) from *Escherichia coli* O111: B4, sodium 3’-[1-(phenylaminocarbonyl)-3,4-tetrazolium]-bis(4-methoxy6-nitro) benzene sulfonic acid hydrate (XTT), N-methyl dibenzopyrazine methyl sulfate (PMS) were obtained from Sigma-Aldrich (St. Louis, MO, USA). The 2x qPCRBIO SyGreen 1step Lo-ROX was obtained from PCR Biosystems (Wayne, PA, USA). Tri-RNA Reagent was purchased from Favorgen (Kaohsiung, Taiwan). IL-6 and PGE_2_ quantitative sandwich ELISA kits were obtained from Abcam (Cambridge, MA, USA). The Griess reagent was purchased from Promega (Madison, WI, USA). All other reagents were obtained from Sigma-Aldrich unless otherwise described

### 4.2. Plant Materials and Preparation of the Extracts

Leaves of *O. tenuiflorum* were cleaned with distilled water twice. Then, 500 g of sample was dried at 60 °C and then powdered into fine powder. Two liters of 70% ethanol were added to powdered dried plants and allowed to remain for 24 h at room temperature. The mixture was filtered and evaporated using a rotary evaporator to eliminate the ethanol. The liquid was subsequently freeze-dried and stored at −20 °C until used.

### 4.3. Phytochemical Analyses

The phytochemical profile was analyzed using liquid chromatography-electrospray ionization tandem mass spectrometry (LC-ESI-MS/MS) with the conditions and validation following a well-established protocol as previously reported [56] without any modification. The LC–ESI-MS/MS system consisted of an ultrahigh-performance liquid chromatography (UHPLC) system (a Dionex Ultimate 3000) attached to a mass spectrometer (TSQ Quantis Triple Quadrupole) and a diode array detector (DAD) from Thermo Fisher Scientific (Bremen, Germany). A separation of phenolics was performed on an Accucore RP-MS column (2.1 mm × 100 mm, 2.6 μm, Thermo Fisher Scientific, Bremen, Germany) with a gradient mobile phase (solvent A: acetonitrile and solvent B: Milli-Q water (18.2 MΩ·cm resistivity at 25 °C) containing 0.1% (*v*/*v*) formic acid). Authentic standards consisted of syringic acid (>97.0% T), sinapic acid (>99.0% GC, T), quercetin (>98.0% HPLC, E), naringenin (>93.0% HPLC, T), myricetin (>97.0% HPLC), luteolin (>98.0% HPLC), kaempferol (>97.0% HPLC), hydroxybenzoic acid (>99.0% GC, T), 4–hesperidin (>90.0% HPLC, T), genistein (>98.0% HPLC), ferulic acid (>98.0% GC, T), (−)-epigallocatechin gallate (>98.0% HPLC), 3,4-dihydroxybenzoic acid (≥97% T), *p*-coumaric acid (>98.0% GC, T), cinnamic acid (>98.0% HPLC), chlorogenic acid (>98.0% HPLC, T), caffeic acid (>98.0% HPLC, T) and apigenin (>98.0% HPLC) from Tokyo Chemical Industry (Tokyo, Japan), vanillic acid (≥97% HPLC), rutin (≥94% HPLC), rosmarinic acid (≥98% HPLC) and gallic acid (97.5–102.5% T) from Sigma-Aldrich (St. Louis, MO, USA), galangin (≥98.0% HPLC) from Wuhan ChemFaces Biochemical Co., Ltd. (Hubei, China) and isorhamnetin (≥99.0% HPLC) from Extrasynthese (Genay, France). The LC–ESI-MS/MS chromatograms were shown in Figure 1.

### 4.4. Bacterial Culture and Identification

Three isolates of each of *S. aureus*, *S. agalactiae*, CNS, and *E. coli* from clinical bovine mastitis cases were obtained from milk samples and collected from the Dairy Farming Promotion Organization of Thailand (D.P.O.), Saraburi, Thailand. Microorganism isolates were characterized based on their culture, morphological (Gram stain), and biochemical properties and sub-cultured on the selective medium following standard microbiological techniques [57]. The standard strains of bacteria used in this study were *S. agalactiae* (ATCC17129), *S. aureus* (ATCC25923), and *E. coli* (ATCC25922). All strains were cryopreserved at −80 °C and maintained in sterile Mueller Hinton Broth (MHB) containing 10% (*v*/*v*) glycerol.

### 4.5. Antibacterial Drugs and Extract of O. tenuiflorum

Working solutions of extracts were prepared by adding 250 μL of dimethyl sulfoxide (DMSO, Sigma-Aldrich, St. Louis, MO, USA) to 50 mg of a lyophilized extract of *O. tenuiflorum*, followed by the addition of 1750 μL of distilled water to a final concentration of 50 mg/mL in 25% DMSO. The solution was filtered through a 0.45 μm pore size membrane to prevent contamination from other bacteria. Antibacterial drugs such as penicillin, amoxicillin-clavulanic acid (4:1 combination), cefazolin, and gentamicin obtained from Sigma-Aldrich (St. Louis, MO, USA) were evaluated for use in the treatment of bovine mastitis in Thailand. Working solutions were prepared in PBS with a unique concentration of each antibacterial.

### 4.6. In Vitro Determination of Antibacterial Susceptibility

Minimum inhibitory concentration (MIC) and minimal bactericidal concentration (MBC) were established using the broth microdilution technique according to the Clinical and Laboratory Standards Institute [42,58] recommendations. The microorganisms were initially grown on brain heart infusion (BHI) agar plates and pre-incubated for 24 h at 37 °C. Single colonies were selected and sub-cultured overnight on MHB. The inoculum was adjusted to 1.5 × 10^8^ CFU/mL using a 0.5× McFarland turbidity standard (OD_600_ = 0.10). Microplate wells were filled with 100 µL of MH broth containing antibacterial drugs and *O. tenuiflorum* extract and 100 µL of a 10^6^ CFU/mL bacterial suspension to achieve the following final concentrations: penicillin, 0.016–64 µg/mL; amoxicillin-clavulanic acid, 0.0625–128 µg/mL; cefazolin, 0.125–256 µg/mL; gentamicin, 1–2048 µg/mL and plant extract, 0.5–1000 µg/mL. The final DMSO concentration in the solutions as 0.5% (*v*/*v*) was used as the negative control. A medium with no inoculum was applied to control sterility. After a 24 h incubation period at 37 °C, 10 µL of 0.5% triphenyltetrazolium chloride (TCC) was applied to each well and incubated for 1 h. The MIC was defined as the lowest concentration that produced no color change and completely inhibited growth [59].

Minimum bactericidal concentration (MBC) was determined following the MIC assay. MBC was defined as the minimum bacterial concentration required to completely kill the original inoculums. Aliquots of 10 μL of supernatants from the wells containing antibacterial drugs or plant extract at the MIC values and higher concentrations were spread onto tryptic soy agar (TSA) plates. The agar plates were incubated at 37 °C for 24 h. MBC was estimated as the least sample concentration where no visible growth was observed on the nutrient agar medium. All MIC and MBC assays were carried out in triplicate in three different experiments.

### 4.7. Evaluation of Synergistic Effect

Evaluation of the interaction between antibacterial drugs and *O. tenuiflorum* extract was performed using the checkerboard broth microdilution method to determine the MIC value of the extract in combination with antibacterial drugs [60,61]. The fractional inhibitory concentration (FIC) index was used to quantify synergistic interactions between the plant extract and antibacterial drugs against important bacterial mastitis pathogens including *S. aureus*, *S. agalactiae* and CNS. Bacterial cultures were grown in *O. tenuiflorum* extract in the presence of penicillin, amoxicillin-clavulanic acid, cefazolin, and gentamicin at concentrations ranging from 1/8 MIC to 4MIC. These studies were carried out in the same way as the MIC determination in susceptibility testing. The fractional inhibitory concentration (FIC) index was used to determine the antibacterial impact of each combination using Equation (1):

Fractional inhibitory concentration (FIC) index = ΣFIC = FIC (antibacterial) + FIC (plant extract),(1)

where FIC (antibacterial) = MIC of antibacterial in combination/MIC of antibacterial alone, and FIC (extract) = MIC of extract in combination/MIC of extract alone. Interactions were categorized as synergistic for ΣFIC values of ≤0.5, additive (≥0.5–1.0), indifferent (≥1.0–≤4.0) or antagonistic (ΣFIC > 4.0) [61].

### 4.8. Cell Culture

A mouse macrophage cell line (RAW 264.7) was obtained from the American Type Culture Collection (ATCC #TIB-71, Manassas, Virginia, USA). Cells were cultured in Dulbecco’s modified Eagles’ medium (DMEM), which contained 10% fetal bovine serum (FBS), 100 IU/mL penicillin, and 100 µg/mL streptomycin, and were incubated at 37 °C with 5% CO_2_. The cells were maintained in 25 cm^2^ flasks and passaged when they achieved 80% confluence. The medium, FBS, L-glutamine, penicillin, streptomycin, fungizone, and trypsin were acquired from GIBCO (Waltham, MA, USA).

### 4.9. Cytotoxicity Assay with RAW 264.7 Cells

Cell viability of RAW 264.7 macrophages was assessed using the sodium 3´-[1- (phenylaminocarbonyl)- 3,4-tetrazolium]-bis (4-methoxy-nitro) benzene sulfonic acid hydrate (XTT)-based assay to determine the concentration of *O. tenuiflorum* extract that was non-toxic to cells. Cells were seeded in 96-well plates at 1 × 10^5^ cells/well in DMEM containing 10% FBS and cultured overnight. The cells were then treated with 0.5% DMSO (Sigma-Aldrich, St. Louis, MO, USA), which served as a control, or *O. tenuiflorum* extract (15.625, 31.25, 62.5, 125, 250, 500, 1000 or 2000 µg/mL) for another 24 h. Cytotoxicity by XTT reduction assay followed the method described previously, with results quantified as a percentage of the control [62].

### 4.10. Anti-Inflammatory Activity Determination

RAW 264.7 cells were cultured overnight in 96-well plates (1 × 10^5^ cells/well) or 6-well plates (1 × 10^6^ cells/well) and then pretreated for 12 h with media containing different concentrations of *O. tenuiflorum* extract (5, 25, 50, 100 or 500 µg/mL) before stimulation with LPS (10 ng/mL) for 24 h. Culture supernatants were collected from 96-well plates for interleukin-6 (IL-6), nitric oxide (NO), and prostaglandin E2 (PGE2). Cells were harvested from the 96 or 6-well plates for qPCR analysis.

### 4.11. Measurement of Nitric Oxide (NO) Concentration

Nitric oxide generation was assessed by measuring the quantity of nitrite in the culture medium based on the Griess reaction [63]. Following the experiments, the cell culture supernatant was collected and mixed in a 1:1 (*v*/*v*) ratio with Griess reagent (1% sulfanilamide, 5% phosphoric acid, and 0.1% N-(1-naphthyl)-ethylenediamine). After incubation for 10 min at room temperature, absorbance was measured at 540 nm using a microplate reader (Biotek, Winooski, VT, USA). A nitrite standard curve was used to calculate nitrite concentration in the supernatant.

### 4.12. Reverse Transcription-Quantitative Polymerase Chain Reaction (RT-qPCR) Analysis for Proinflammatory Gene Expression

To investigate the effects of *O. tenuiflorum* extract on LPS-induced RAW 264.7 cell inflammation, the relative mRNA levels of IL-6, tumor necrosis factor-α (TNF-α), interleukin-1β (IL-1β), inducible nitric oxide synthase (iNOS) and cyclooxygenase-2 (COX-2) were measured by RT-qPCR. Total RNA was extracted by homogenizing the cells in Tri-RNA Reagent according to the manufacturer’s instructions. The RT-qPCR reaction mixture, qPCRBIO SyGreen 1-step Lo-ROX (PCR Biosystems, Wayne, PA, USA), was made according to the manufacturer’s guidelines. Quantitative polymerase chain reaction (qPCR) was performed by qTOWER3 Real-Time PCR Systems (Analytik Jena, Langewiesen, Germany). Thermal cycling conditions were used to amplify the target genes using the following parameters: reverse transcription step at 45 °C for 10 min, polymerase activation step at 95 °C for 2 min, denaturation step at 40 amplification cycles at 95 °C for 5 s, and the final step at 60 °C for 30 s. Relative levels of gene expression were normalized to glyceraldehyde 3-phosphate dehydrogenase (GAPDH) mRNA using the 2^−ΔΔCT^ method [64]. The primer sequences are listed in Table 2.

### 4.13. Determination of IL-6 and PGE2 Productions by Enzyme-Linked Immunosorbent Assay

The levels of IL-6 and PGE2 in RAW 264.7 cell culture supernatants were measured using an enzyme-linked immunosorbent assay (ELISA) kit obtained from Abcam (Cambridge, MA, USA) and performed according to the manufacturer’s instructions.

### 4.14. Statistical Analysis

Statistical analysis was performed using GraphPad Prism ver. 5 software. The values are presented as mean ± standard deviation (SD) from at least three independent replicates. Statistical significance between groups was determined using a one-way ANOVA followed by Tukey’s test. Statistical significance between groups was determined using a one-way ANOVA followed by Tukey’s test. A probability of 0.05 or less (*p* ≤ 0.05) was considered statistically significant.

## 5. Conclusions

This study explored new antibacterial candidates from plants with multi-target abilities in mastitis treatment, covering antibacterial and anti-inflammation activities. Results demonstrated promising anti-mastitis properties of *O. tenuiflorum* ethanolic extract as follows: (i) *O. tenuiflorum* had antibacterial effects against Gram-positive bacteria including *S. aureus*, CNS, and *S. agalactiae* but not Gram-negative bacteria, (ii) *O. tenuiflorum* extract showed synergistic effects with penicillin or amoxicillin-clavulanic acid against all tested strains, while cefazolin and amikacin had an additive effect, and (iii) *O. tenuiflorum* extract showed anti-inflammatory activities, with reduced expression of inflammatory molecules in LPS-treated macrophages. However, further investigations on route and dosage for therapy in animal models are required.

## Figures and Tables

**Table 1:** Minimal inhibitory concentrations (MICs) and minimal bactericidal concentrations (MBCs) of antibacterial drugs and *O. tenuiflorum* leaf extract against important bacterial mastitis pathogens.

Bacterial Strains *	Penicillin		Amoxicillin-Clavulanic Acid		Cefazolin		Gentamicin		*O. tenuiflorum* Leaves Extract	
	MIC (µg/mL)	MBC (µg/mL)	MIC (µg/mL)	MBC (µg/mL)	MIC (µg/mL)	MBC (µg/mL)	MIC (µg/mL)	MBC (µg/mL)	MIC (µg/mL)	MBC (µg/mL)
*S. aureus* (ATCC25923)	0.25	1	0.0625	0.125	4	16	4	32	15.6	62.5
*S. aureus* (CI)	16	128	4	16	64	256	16	128	31.2	125
*CNS* (CI)	0.125	1	8	64	1	8	16	128	3.9	15.6
*S. agalactiae* (ATCC17129)	0.5	16	0.0625	8	4	128	32	128	7.8	125
*S. agalactiae* (CI)	2	32	1	32	32	256	64	256	31.2	500
*E. coli* (ATCC25922)	-	-	16	32	4	128	2	16	>1000	-
*E. coli* (CI)	-	-	32	128	16	256	2	16	>1000	-

* CI = clinical isolate.

**Table 2:** Primers used for real-time PCR.

Genes	Primers	Sequences	Reference
GAPDH (mouse)	forward	CTCGTGGAGTCTACTGGTGT	[65]
	reverse	GTCATCATACTTGGCAGGTT	
iNOS (mouse)	forward	ATGAGGTACTCAGCGTGCTCCAC	[66]
	reverse	CCACAATAGTACAATACTACTTGG	
IL-1β (mouse)	forward	CGACAAAATACCTGTGGCCT	[67]
	reverse	TTCTTTGGGTATTGCTTGGG	
IL-6 (mouse)	forward	GGAGGCTTAATTACACATGTT	[65]
	reverse	TGATTTCAAGATGAATTGGAT	
TNF-α (mouse)	forward	TTCTGTCTACTGAACTTCGG	[65]
	reverse	GTATGAGATAGCAAATCGGC	
COX-2 (mouse)	forward	AAGAGCATCGCAGAGGT	[68]
	reverse	CCCATTAGCAGCCAGTT	

## Data Availability

The data used to support the findings of this study are available from the corresponding author upon request.
